# Daptomycin versus linezolid for treatment of vancomycin-resistant enterococcal bacteremia: systematic review and meta-analysis

**DOI:** 10.1186/s12879-014-0687-9

**Published:** 2014-12-13

**Authors:** Yu-Chung Chuang, Jann-Tay Wang, Hsin-Yi Lin, Shan-Chwen Chang

**Affiliations:** Department of Internal Medicine, National Taiwan University College of Medicine and Hospital, 7 Chung-Shan South Road, Taipei, 100 Taiwan; Graduate Institute of Clinical Medicine, College of Medicine, National Taiwan University, Taipei, Taiwan; Department of Economics, National Chengchi University, Taipei, Taiwan

**Keywords:** Daptomycin, Linezolid, Meta-analysis, Vancomycin-resistant enterococci

## Abstract

**Background:**

Linezolid, which has bacteriostatic activity, is approved for the treatment of vancomycin-resistant enterococci (VRE) infections. Meanwhile, daptomycin exerts bactericidal activity against VRE, but is not approved for the treatment of VRE bacteremia. Only a few studies with small sample sizes have compared the effectiveness of these drugs for treatment of VRE bacteremia.

**Methods:**

PubMed, EMBASE, and the Cochrane Library were searched for studies of VRE bacteremia treatment published before January 1, 2014. All studies reporting daptomycin and linezolid treatment outcomes simultaneously were included. The endpoints were mortality and microbiological cure. The adjusted odds ratios (aORs) of mortality in daptomycin- and linezolid-treated patients were extracted if available. Pooled odds ratios (ORs) and 95% confidence intervals (CIs) were calculated for all outcomes using a random-effects model.

**Results:**

Thirteen studies (532 patients receiving daptomycin, 656 patients receiving linezolid) met the selection criteria. All studies had retrospective cohort designs and relatively small sample sizes. Eight studies compared the aORs of mortality in daptomycin- and linezolid-treated patients. Four studies were published as conference papers and there was significant heterogeneity among these studies (*I*^*2*^ = 63%, *p* = 0.04). Daptomycin use was not associated with better microbiological cure (daptomycin *vs*. linezolid, OR: 0.67, 95% CI: 0.42–1.06, *p =* 0.09). However, mortality was higher in patients receiving daptomycin (OR: 1.43, 95% CI: 1.09–1.86, *p =* 0.009). Subgroup analysis of studies that reported aORs indicated that daptomycin was associated with higher mortality (OR: 1.59, 95% CI: 1.02–2.50, *p =* 0.04). There was no evidence of publication bias, but all enrolled studies were retrospective, had small sample sizes, and had substantial limitations.

**Conclusions:**

Although limited data is available, the current meta-analysis shows that linezolid treatment for VRE bacteremia was associated with a lower mortality than daptomycin treatment. However, the results should be interpreted cautiously because of limitations inherent to retrospective studies and the high heterogeneity among studies. A large randomized trial is needed to confirm the present results.

**Electronic supplementary material:**

The online version of this article (doi:10.1186/s12879-014-0687-9) contains supplementary material, which is available to authorized users.

## Background

Vancomycin-resistant enterococci (VRE) were first reported in 1986 [1],[2], and since then have become increasingly responsible for hospital-acquired infections, especially in intensive care units [3]. Currently, VRE bacteremia is a significant independent predictor of mortality in patients with enterococcal bloodstream infections (BSIs) [4]. Current treatment options are limited [5].

Linezolid, an oxazolidinone, is approved for treatment of VRE infection but there are concerns about its use for treatment of VRE bacteremia because it can suppress bone marrow, it has bacteriostatic not bacteriocidal activity, and resistant VRE strains have been reported [6],[7]. Daptomycin, a cyclic lipopeptide, exhibits rapid bactericidal activity against VRE [8] and has been successfully used to treat VRE bacteremia [9]-[11]. Daptomycin is not approved for the treatment of VRE bacteremia. Emerging studies suggest daptomycin may have activity similar to that of linezolid [12]-[19]. However, all of these studies had small sample sizes and insufficient statistical power to compare the efficacy of these drugs in treatment of VRE bacteremia.

A large randomized controlled trial is the best method to compare the efficacy of daptomycin and linezolid in treatment of VRE bacteremia. However, such a trial was halted prematurely because of logistic challenges [20]. A search of the clinical trial registration database (www.clinicaltrials.gov) indicated that there were no ongoing trials comparing daptomycin and linezolid for the treatment of VRE bacteremia. Recent systematic reviews and meta-analyses showed that there was a tendency for linezolid to provide better survival than daptomycin [21],[22]. However, possible confounders were not adjusted for in the meta-analysis by Whang et al. [21]. In addition, these meta-analyses included conference abstracts as well as full papers [21],[22]. Although inclusion of conference papers might reduce publication bias, there may be differences in the results reported in conference abstracts and subsequent publications [23]. In addition, several recent studies [17],[19],[24] were not cited in these meta-analyses [21],[22].

The purpose of the present study was to determine whether daptomycin is as effective as linezolid for treatment of VRE bacteremia. We systematically reviewed the literature on the effects of daptomycin and linezolid upon the clinical outcomes of patients with VRE bacteremia and synthesized all available data into a meta-analysis. In order to control for possible confounders in each study, we extracted the adjusted effect estimates in analysis of mortality. We also examined the impact of statistical adjustment of effect estimates, and whether the studies were full papers or conference papers.

## Methods

We followed the recommendations of the Preferred Reporting Items for Systematic Reviews and Meta-Analyses (PRISMA) [25]. No protocol of the present review was previously published or registered.

### Literature search

We searched PubMed, EMBASE, the Cochrane Library, and ClinicalTrials.gov for relevant articles up to January 1, 2014. The following search terms were applied to articles published since January 1950: *Enterococcus* AND (infection OR bacteremia) AND (linezolid OR daptomycin) AND vancomycin resistant. The literature search was limited to English-language publications of human subjects. We also reviewed the abstracts from the annual meetings of the Infectious Disease Society of America and the Interscience Conference on Antimicrobial Agents and Chemotherapy. The references of 7 review articles on treatments for VRE infections were examined to identify additional studies not found in the computerized databases [5],[7],[21],[22],[26]-[28].

### Study selection

All included studies were clinical trials or observational studies of the treatment of patients with VRE bacteremia that reported daptomycin and linezolid treatment outcomes simultaneously. Epidemiology studies that didn’t report daptomycin and linezolid treatment outcomes were excluded. Prophylaxis studies and studies lacking clinical endpoint data were excluded. Study quality was assessed using SIGN50, and studies with unacceptable quality were excluded [29].

### Data extraction, definitions, and outcomes

Two physician reviewers (Y.-C.C. and J.-T.W.) independently evaluated each study and abstracted the following: study characteristics (design, country, time period), patient population (number of evaluated patients, disease severity, underlying comorbidities, presence of infective endocarditis), antibiotic usage (type, dosage), and adverse events (anemia, leukopenia, thrombocytopenia, acute kidney injury [AKI], elevated creatinine kinase [CK]).

The primary outcome was mortality. Clinical cure and microbiological cure rate, as assessed by the investigators of each study, were also recorded. Mortality was classified as long-term (30 days, and overall in-hospital mortality) or short-term (14 days, mortality at the end of therapy [EOT], mortality within 7 days after EOT, and infection-related mortality). For 8 studies, we also extracted and compared the adjusted odds ratios (aORs) of mortality in patients treated with daptomycin and linezolid. These studies adjusted for possible confounders, such as underlying disease and disease severity.

### Statistical analysis

Pooled odds ratios (ORs) and 95% confidence intervals (CIs) of all outcomes were calculated using the DerSimonian–Laird random effects model. Heterogeneity was estimated from the inverse-variance fixed-effect model. Statistical heterogeneity among studies was assessed by the *χ*^2^ test (*p* < 0.10 was defined as indicating significant heterogeneity) and calculation of *I*^2^. Publication bias was assessed by use of a funnel plot and the Egger test. Univariate meta-regression analyses were performed to examine the impacts of a reported aOR and publication type on the results of the meta-analysis. All statistical analyses were performed using STATA version 12 (StataCorp, College Station, TX, USA) and Review Manager 5.2. (The Nordic Cochrane Centre, Copenhagen, Denmark).

## Results

### Characteristics of included studies

**Figure 1:**
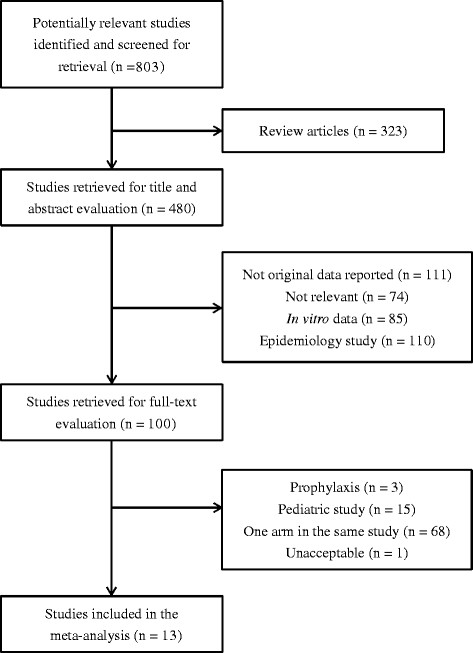
**Flow diagram of the systematic search and study selection process.**

A search of the 3 databases led to the initial identification of 803 articles, 13 of which were ultimately included in the analysis [12]-[19],[24],[30]-[33] (Figure 1). According to the SIGN50 criteria, none of the 13 studies were classified as high quality. The study by Weinstock et al. [34] (Figure 1) was classified as unacceptable quality and excluded since that the study didn’t clearly define the outcome and the exposure, which might result in detection bias. All enrolled studies were classified as acceptable quality with some potential flaws in each study with an associated risk of bias. The aORs of mortality for daptomycin vs. linezolid treatment were extracted from our previously published cohort study [24]. Raw data from Chou et al. [17] were retrieved by email communication for calculation of aORs.

Additional file 1: Table S1 and S2 describe the general and detailed characteristics of the 13 eligible studies, all of which were retrospective cohort studies with relatively small sample sizes [12]-[19],[24],[30]-[33]. Two studies were performed at multiple institutions [12],[13] and 3 studies focused on hematologic or neutropenic populations [14],[19],[33]. Two studies were conducted in Taiwan [17],[24] and the others were conducted in the USA. Four studies were published as conference papers [30]-[33]. All studies reported mortality. Eight studies reported microbiological outcomes [12],[15],[16],[18],[30]-[33]. Eight studies reported long-term mortality [12]-[14],[16]-[19],[33]; one study reported 14-day mortality [24]; two studies reported mortality at EOT [30],[32]; one study reported mortality within 7 days after EOT [15]; and one study reported infection-related mortality [31]. Eight studies compared the aORs of mortality in daptomycin- and linezolid-treated patients [12],[13],[15]-[17],[24],[30],[32]. These eight studies used multivariate analysis to adjust for factors such as age, sex, Charlson comorbidity index, thrombocytopenia, timing of antibiotics, intensive care unit stay, and disease severity (e.g., APACHE II score, and shock) [12],[13],[15]-[17],[24],[30],[32]. Only 5 studies reported *a priori*-defined adverse events [12],[14],[15],[19],[31].

### Meta-analysis results

#### Mortality

**Figure 2:**
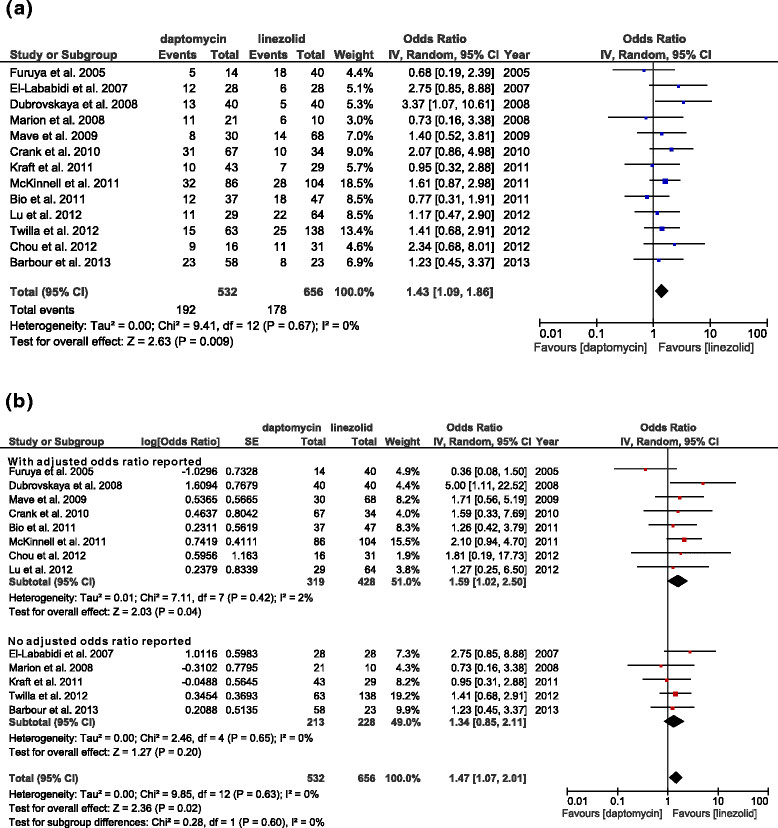
**Forest plots of raw mortality and mortality in which aORs were and were not reported. (a)** ORs of all 13 studies. **(b)** ORs of studies that did (n = 8) and did not (n = 5) report aORs. Here and below, the vertical line indicates the “no difference” point of the 2 regimens and the horizontal lines indicate 95% confidence intervals (CIs). ∎, odds ratio; ♦, pooled odds ratio for all studies.

**Figure 3:**
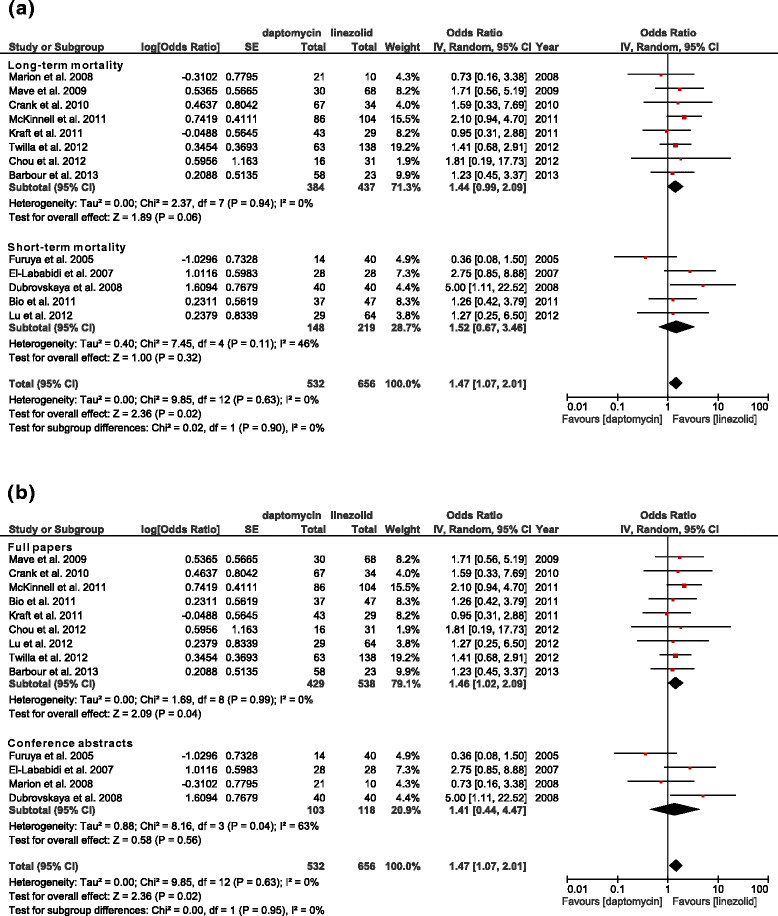
**Forest plots of long-term and short term mortality and of full papers and conference abstracts. (a)** ORs of studies that reported long-term (n = 8) and short-term (n = 5) mortality. **(b)** ORs of studies that were full papers (n = 9) and conference abstracts (n = 4).

Overall, the 13 studies had data that compared the relative rates of raw mortality of 1188 patients who were treated with daptomycin (n = 532) or linezolid (n = 656) (Figure 2a). The results indicate that daptomycin was associated with significantly higher mortality (OR: 1.43, 95% CI: 1.09–1.86, *p =* 0.009, *I*^2^ = 0%). However, there were baseline differences of the 2 study groups, the aORs for mortality in daptomycin- and linezolid-treated patients were analyzed in further detail . We also performed 3 subgroup analyses of these studies. In particular, we analyzed studies in which aORs were reported or not (Figure 2b); studies in which long-term and short-term mortality were reported (Figure 3a); and studies that were published as full papers or conference abstracts (Figure 3b).

Among studies that reported aORs, daptomycin was associated with significantly higher mortality (Figure 2b, 747 patients, OR: 1.59, 95% CI: 1.02–2.50, *p =* 0.04, *I*^2^ = 2%). However, among studies that did not report aORs, daptomycin was not associated with significantly higher mortality (Figure 2b, 441 patients, OR: 1.34, 95% CI: 0.85–2.11, *p =* 0.20, *I*^2^ = 0%). There was no significant difference between two subgroups (*p =* 0.54).

Patients who received daptomycin had borderline significantly higher long-term mortality (Figure 3a, 821 patients, OR: 1.44, 95% CI: 0.99–2.09, *p =* 0.06, *I*^2^ = 0%), but not significantly short-term mortality (367 patients, OR: 1.52, 95% CI: 0.67–3.46, *p =* 0.32, *I*^2^ = 46%).

Published full papers showed that patients who received daptomycin had higher mortality (Figure 3b, 967 patients, OR: 1.46, 95% CI: 1.02–2.09, *p =* 0.04, *I*^2^ = 0%). Conference abstracts indicated no significant mortality differences, and there was significant heterogeneity among these studies (221 patients, OR: 1.41, 95% CI: 0.44–4.47, *p =* 0.56, *I*^2^ = 63%).

#### Meta-regression analysis

Univariate meta-regression analyses indicated that the results of the meta-analysis were not significantly affected by reported OR (aOR vs. OR, *p =* 0.556), publication type (conference abstract vs. full paper, *p =* 0.948), or outcome definition (long-term vs. short-term mortality, *p* = 0.842).

#### Publication bias

**Figure 4:**
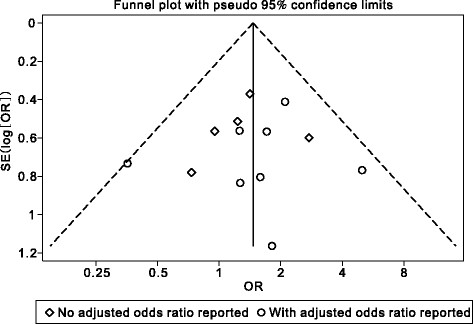
**Funnel plot showing the absence of publication bias.**

A funnel plot of the 13 included studies indicates no significant publication bias with respect to mortality (Figure 4, slope coefficient = 0.574, *p =* 0.298). In addition, the results of the test for small study effects indicated that this was not significant (*p =* 0.710).

#### Clinical outcome, microbiological outcome, and relapse

**Figure 5:**
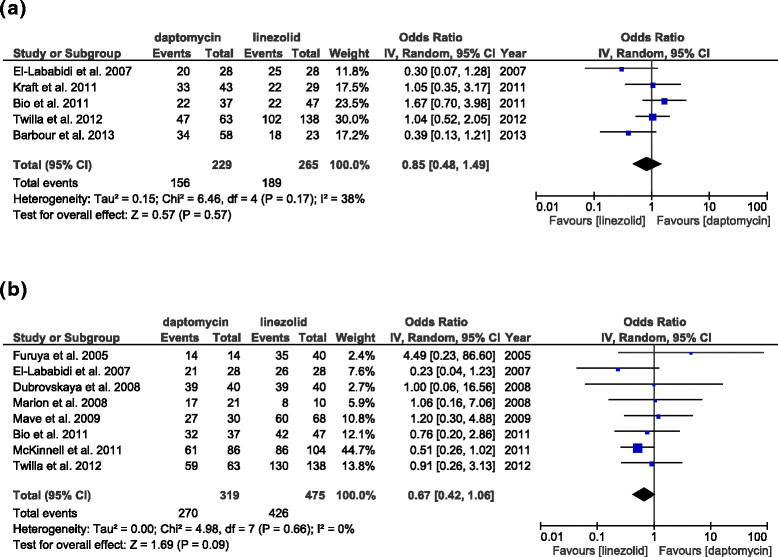
**Forest plots of studies that reported clinical cure and microbiological cure. (a)** ORs of studies that reported clinical cure (n = 5). **(b)** ORs of studies that reported microbiological cure (n = 8).

**Figure 6:**
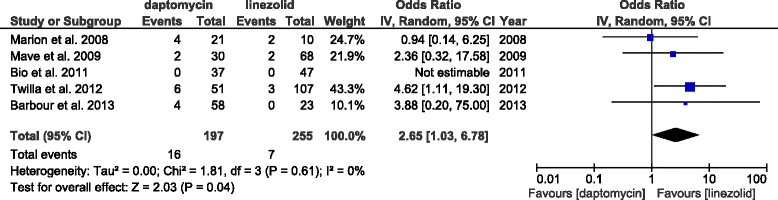
**Forest plots of studies that reported relapse.** ORs of studies that reported relapse (n = 5).

Five studies (494 patients) reported clinical cure rates. The results indicate no significant difference between linezolid and daptomycin treatment (Figure 5a, daptomycin vs. linezolid, OR: 0.85, 95% CI: 0.48–1.49, *p =* 0.57, *I*^2^ = 38%). Eight studies (794 patients) reported microbiological cure rates. Linezolid treatment had a trend for better microbiological cure (Figure 5b, daptomycin vs. linezolid, OR: 0.67, 95% CI: 0.42–1.06, *p =* 0.09, *I*^2^ = 0%). Five studies (452 patients) reported relapses of VRE bacteremia. The results indicate that daptomycin use was associated with significantly higher relapse rates (Figure 6, daptomycin vs. linezolid, OR: 2.65, 95% CI: 1.03–6.78, *p =* 0.04, *I*^2^ = 0%).

#### Adverse events

Five studies reported *a priori*-defined adverse events. Hematologic adverse events could not be analyzed, because of differences in the reporting criteria. Although 3 studies reported that daptomycin was associated with trends toward lower rates of thrombocytopenia (pooled OR: 0.41, 95% CI: 0.14–1.18, *p =* 0.10, *I*^2^ = 0%) [12],[15],[31], the other study reported that the linezolid group had a shorter duration of thrombocytopenia (15.7 vs. 18.0 days) [14]. Two studies (170 patients) provided data comparing the relative rates of AKI due to daptomycin or linezolid [12],[14]. The results indicate that daptomycin was not associated with a significant risk of AKI (pooled OR: 1.59, 95% CI: 0.49–5.14, *p =* 0.44, *I*^2^ = 0%). Three studies (254 patients) had data comparing the relative rates of elevated CK [12],[14],[15]. Again, daptomycin was not associated with significant risk of elevated CK (pooled OR: 1.97, 95% CI: 0.37–10.46, *p =* 0.43, *I*^2^ = 0%) [15],[24],[30]-[32].

## Discussion

Thirteen studies compared daptomycin and linezolid for the treatment of VRE bacteremia, and these were all relatively small retrospective cohort studies. Although daptomycin has bactericidal activity against enterococci [8], the present results surprisingly show daptomycin was not associated with significantly better microbiological cure rate than linezolid. In fact, consideration of all 13 studies in the meta-analysis indicated that daptomycin was associated with significantly higher mortality than linezolid. In addition, subgroup analysis of studies that reported aORs also showed linezolid treatment had a more favorable outcome.

Recent meta-analyses by Whang et al. [21] and Balli et al. [22] indicated trends for increased survival with linezolid compared to daptomycin in treatment of VRE infections (OR: 1.3, *p* = 0.054 and OR: 1.41, *p* = 0.02, respectively). There are some limitations of these meta-analyses. Whang et al. [21] did not adjust for possible confounders, so they may have used inaccurate ORs. Balli et al. [22] reported that the daptomycin group had higher mortality, and they confirmed this result when aORs were pooled. It is noted that Furuya et al. [30] reported the adjusted OR in their original paper. However, Balli et al. [22] mis-classified Furuya et al. [30] into the group of unadjusted OR. If this mis-classification is corrected in the meta-analysis by Balli et al., [22] then daptomycin group did not have higher mortality when aORs were pooled in their paper.

Furthermore, these 2 previous meta-analyses included conference abstracts as well as full papers. Although inclusion of conference papers might result in less publication bias, there might be major differences in the results and conclusions of conference abstracts and the subsequent full papers [23]. Although we found no significant differences in the results of full papers and conference abstracts, there was high heterogeneity among conference abstracts (*I*^*2*^ = 62%). Furthermore, these conference abstracts were missing important information, such as outcome definitions and enrollment criteria. In general, the information provided by conference papers is limited, making it difficult to evaluate study quality [29].

Our results do not necessarily exclude the possibility that bactericidal agents may be associated with better outcomes [35]. In particular, various criteria are used to define bactericidal effects *in vitro*, but such definitions can be somewhat arbitrary in clinical settings [35]. Enterococci are inherently less virulent organisms and generally infect immunocompromised patients. In fact, most infected patients have high Charlson comorbidity index scores [12],[15]-[17]. Under such clinical settings, it is even more debatable whether bactericidal agents are better than bacteriostatic agents.

All of the 13 studies in our meta-analysis were relatively small retrospective cohort studies. Thus, there are several important limitations.

First, daptomycin exhibits rapid concentration-dependent bactericidal activity *in vitro* against Gram-positive organisms, including enterococci [36]. Therefore, the daptomycin dosage and minimum inhibitory concentration (MIC) against specific VRE should be considered in outcome analyses. A previous case series noted better outcomes for patients given a daptomycin dosage of more than 6 mg/kg/day [11] and four studies of daptomycin dosage reported a mean or median dosage of 6 mg/kg/day [12],[13],[15],[18]. However, different daptomycin dosages were used in four studies included in the present meta-analysis (4.5–6 mg/kg/day [14], 3.7–8.8 mg/kg/day [15], 3.4–10.4 mg/kg/day [18], and 4–9 mg/kg/day [32]) and another four studies did not mention daptomycin dosage [16],[17],[24],[31]. Therefore, there may have been under-dosing of daptomycin in some patients. In contrast, linezolid was administered consistently at 600 mg q12h [12]-[15],[18],[19],[33]. In addition, only 6 studies reported MICs [12],[14],[15],[18],[19],[24], so the susceptibilities of the VRE isolates to daptomycin and linezolid were not investigated thoroughly.

Second, the time from bacteremia onset to initiation of daptomycin or linezolid treatment was about 2 to 3 days [14]-[19]. However, in cases of septic shock, a delay in administration of an effective antimicrobial agent by only a few hours can decrease survival [37]. Thus, there could have been an underestimate of the effectiveness of daptomycin or linezolid treatment. It is also possible that patients who survived for 2 to 3 days after bacteremia onset until drug administration might have been healthier to begin with. Thus the true effectiveness of daptomycin or linezolid treatment might be over-estimated.

Third, we used the hard endpoint of “mortality” to reduce the likelihood of misclassification bias, but the different studies used different definitions of mortality. Most of the enrolled studies reported long-term mortality [12]-[14],[16]-[18],[33], but five studies reported short-term mortality [15],[24],[30]-[32]. Fourteen-day mortality is suggested for evaluating treatment outcome because this endpoint can reduce potential bias due to the assignment of cause of death, which can be problematic [38]. One of the included studies reported that treatment and microbiological factors affected 14-day morality but not 28-day mortality [24]. This might be because enterococci generally infect compromised hosts with multiple comorbidities, and that these comorbidities may be more significant causes of death. Some of the included studies reported outcomes such as clinical or microbiological cure. However, since all the studies were retrospective, studies that report clinical cure might suffer from recall bias or misclassification bias. There were no pre-defined schedules for following blood cultures, so results regarding microbiologic cure are hard to interpret.

Fourth, there were various confounders among the included studies. For example, patients in the daptomycin groups had significantly higher rates of neutropenia [16] and thrombocytopenia [15],[17], whereas patients in the linezolid groups were significantly older [12],[18]. Meta-analysis of the aORs of these 8 studies continued to favor linezolid. Though these studies tried to adjust the confounders by using multivariate logistic regressions, there are still residual confounding factors [39]. The confounding by indications might result in difficulties in comparing the treatment efficacies in such critical patients in nonrandomized studies. In addition, though all studies reported that there are at least one set of blood culture yielded VRE, however, important information such as the foci of the bacteremic infection and the associated therapy, such as catheter removal or not were not clearly stated. Disease severity is an important factor when evaluating treatment response. However, different studies used different disease severity scores. Although this can be adjusted for in individual studies, it is difficult to analyze the impact of disease severity when combining studies.

Adverse events, especially bone marrow suppression, are another concern regarding the use of linezolid to treat VRE bacteremia. The use of different definitions of hematologic adverse events prevented us from pooling and analyzing this data. However, the linezolid groups apparently did not have a higher rate of adverse hematologic events such as anemia, leukopenia, and thrombocytopenia. In fact, an analysis of phase III trials showed that linezolid was no more likely to cause adverse effects than the drugs to which it was compared [40]. Other research indicated that thrombocytopenia and a slightly increased risk of anemia occurred after 2 or more weeks of linezolid treatment [41]. However, most of the studies examined in the present meta-analysis administered linezolid for only 10–14 days [14],[15],[18]. We also found no significant evidence that daptomycin results in a higher incidence of CK elevation than linezolid (pooled OR: 1.97, *p =* 0.43).

## Conclusions

Although limited data is available, the current meta-analysis shows that linezolid treatment for VRE bacteremia results in lower mortality than daptomycin treatment. However, this should be interpreted cautiously because of the limitations inherent to the retrospective studies in this meta-analysis. Rather than concluding linezolid is superior to daptomycin based on this meta-analysis, we strongly recommend a large randomized trial with adequate dosages to validate this result.

## Additional file

## Electronic supplementary material

Additional file 1: Table S1.: General characteristics and results of the 13 studies included in the meta-analysis. Table S2. Detailed characteristics of the 13 studies included in the meta-analysis. (DOCX 31 KB)

Below are the links to the authors’ original submitted files for images.

Authors’ original file for figure 1

Authors’ original file for figure 2

Authors’ original file for figure 3

Authors’ original file for figure 4

Authors’ original file for figure 5

Authors’ original file for figure 6

## Abbreviations

AKI: Acute kidney injury
aOR: Adjusted odds ratio
BSI: Blood stream infection
CI: Confidence interval
CK: Creatinine kinase
MIC: Minimum inhibitory concentration
OR: Odds ratio
PRISMA: Preferred reporting items for systematic reviews and meta-analyses
VRE: Vancomycin-resistant entercocci
