# Supplementary material for: Daptomycin versus linezolid for treatment of vancomycin-resistant enterococcal bacteremia: systematic review and meta-analysis
Source: BMC Infect Dis. 2014 Dec 13;14:687. doi: 10.1186/s12879-014-0687-9 (PMC4269951; doi:10.1186/s12879-014-0687-9)
Supplement: Supplementary file 1 — Additional file 1: Table S1.: General characteristics and results of the 13 studies included in the meta-analysis. Table S2. Detailed characteristics of the 13 studies included in the meta-analysis. (DOCX 31 KB) [file 12879_2014_687_MOESM1_ESM.docx]

Additional file 1

Table S1. General characteristics and results of the 13 studies included in the meta-analysis.

|  |  |  | Microbiological Cure |  | Mortality |  | Adverse events  (daptomycin vs. linezolid) | Possible confounders^c^ (daptomycin vs. linezolid) |
| --- | --- | --- | --- | --- | --- | --- | --- | --- |
| Author, Year | Design, Year, Country | No. of patients | Daptomycin,  *n*/*N* (%) | Linezolid,  *n*/*N* (%) | Daptomycin,  *n*/*N* (%) | Linezolid,  *n*/*N* (%) |  |  |
| Mave et al. 2009 [12] | Two-center,  retrospective cohort, Sep. 2003–Dec. 2007,  USA | 98 | 27/30 (90) | 60/68 (88.2) | 8/30 (26.7) | 14/68 (20.6) | Anemia (3 vs. 8), thrombocytopenia (3 vs. 8), AKI (3 vs. 4), Elevated CK (0 vs. 0) | Age  (51 vs. 60 years),  ICU  (53.3% vs. 29.4%) |
| Crank et al. 2010 [13] | Three-center,  retrospective cohort, Sep. 2003–Jun. 2007,  USA | 101 | NR | NR | 31/67 (46.3) | 10/34 (29.4) | NR | Shock  (31.3% vs. 11.8%),  previous vancomycin  (84.8% vs. 52.9%),  previous linezolid use  (31.8% vs. 0%) |
| Kraft et al. 2011 [14] | Single-center, retrospective cohort, Jan. 2004–Jan. 2007,  USA | 72 | NR | NR | 10/43 (23.3) | 7/29 (24.1) | Neutropenia duration (20.3 vs. 21.0), Thrombocytopenia duration (18.0 vs. 15.7), AKI (4 vs. 2), Elevated CK (3 vs. 0) | BMT  (51.2% vs. 18.5%),  AML  (34.5% vs. 65.5%) |
| Bio et al. 2011 [15] | Single-center, retrospective cohort, Jan. 2004–Mar. 2008,  USA | 84 | 32/37 (86.5) | 42/47 (89.4) | 12/37 (32.4) | 18/47 (38.3) | Thrombocytopenia^b^ (1 vs. 7), Elevated CK (2 vs. 2) | ICU  (64.9% vs. 87.2%), Thrombocytopenia (56.8% vs. 19.1%) |
| McKinnell et al. 2011 [16] | Single-center, retrospective cohort, Jan. 2005–Aug. 2008,  USA | 190 | 61/86 (70.9)^a^ | 86/104 (82.7) ^a^ | 32/86 (37.2) | 28/104 (26.9) | NR | Leukopenia  (29% vs. 8%) |
| Chou et al. 2012[17] | Single-center, retrospective cohort, Jan. 2005–Dec. 2010,  Taiwan | 47 | NR | NR | 9/16 (56.3) | 11/31 (35.5) | NR | Mechanical ventilator (68.8% vs. 35.5%),  liver failure  (50% vs. 16.1%),  platelet count  (90.2 vs. 185.0 K/mm^3^) |
| Lu et al. 2012 [24] | Single-center, retrospective cohort, Jan. 2003–Dec. 2010,  Taiwan | 93 | NR | NR | 17/29 (58.6) | 33/64 (51.6) | NR | NR |
| Twilla et al. 2012 [18] | Single-center, retrospective cohort, Jan. 2004–Jul. 2009,  USA | 201 | 59/63 (93.7) | 130/138 (94.2) | 15/63 (23.8) | 25/138 (18.1) | NR | Age  (53vs. 60 years), hematologic malignancy  (33% vs. 14%),  liver transplant  (13% vs. 4%) |
| Barbour et al. 2013 [19] | Single-center, retrospective cohort, Jun. 2005–Jun. 2011,  USA | 81 | NR | NR | 23/58 (39.7) | 8/23 (34.8) | Thrombocytopenia duration (42.2 vs. 51.1) | NR |
| Furuya et al. 2005 [30] | Single-center, retrospective cohort, 2004–2005,  USA | 54 | 14/14 (100) | 35/40 (87.5) | 5/14 (35.7) | 18/40 (45) | NR | NR |
| El-Lababidi et al. 2007 [31] | Single-center, retrospective cohort, Jan. 2000–Dec. 2006,  USA | 56 | 21/28 (75) | 26/28 (92.9) | 12/28 (42.9) | 6/28 (21.4) | thrombocytopenia (0 vs. 4) | Chemotherapy  (46% vs. 14%) |
| Dubrovskaya et al. 2008 [32] | Single-center, retrospective cohort, Jan. 2005–Dec. 2007,  USA | 80 | 39/40 (97.5) | 39/40 (97.5) | 13/40 (32.5) | 5/40 (12.5) | More thrombocytopenia in linezolid group | More hematologic malignancy, neutropenia, renal replacement, and thrombocytopenia in daptomycin group |
| Marion et al. 2008 [33] | Single-center, retrospective cohort, Jun. 2005–Jul. 2007,  USA | 31 | 17/21 (81.0) | 11/21 (52.4) | 8/10 (80) | 6/10 (60) | NR | NR |

^a^ The study reported microbiological failure. Therefore, patients who had no microbiological failure were considered microbiological cures.

^b^ Occurrence of thrombocytopenia in patients who had no thrombocytopenia before treatment.

^c^ Characteristics that were significantly different (*p* < 0.05) between daptomycin- and linezolid-treated groups.

AKI, acute kidney injury; AML, acute myeloid leukemia; BMT, bone marrow transplant; CK, creatinine kinase; ICU, intensive care unit; NR, not reported.

Table S2. Detailed characteristics of the 13 studies included in the meta-analysis

|  |  | Daptomycin | | | | |  | Linezolid | | | | |
| --- | --- | --- | --- | --- | --- | --- | --- | --- | --- | --- | --- | --- |
| Author, Year | Definition of enrolment | Dose | Time*^a^* | MIC | Underlying diseases | Severity |  | Dose | Time*^a^* | MIC | Underlying disease | Severity |
| Mave et al. 2009 [12] | ≥1 positive BC for VRE that met the CDC definition | 6 mg/kg | NR | All susceptible, range 0.25–2 mg/L | CCI >2, 60% | APACHE II median 12.5, range 2–27 |  | 600 mg q12h | NR | All susceptible (≤2 mg/L), but one | CCI >2, 69.1% | APACHE II median 15, range 4–28 |
| Crank et al. 2010 [13] | ≥2 separate positive BC for VRE or 1 positive BC with an identifiable source in a clinical scenario consistent with BSI | mean 5.5, median 6 mg/kg | NR | NR | NR | concomitant shock, 31.3% |  | 600 mg q12h | NR | NR | NR | concomitant shock, 11.8% |
| Kraft et al. 2011 [14] | ≥1 positive BC for VRE, and received linezolid or daptomycin for ≥2 days, hematology or BMT | median 5.5, range 4.5–6 mg/kg | median 1, range 1–2 days | All ≤4 mg/L | NR | NR |  | 600 mg q12h | median 2, range 1–3 days | All ≤2 mg/L | NR | NR |
| Bio et al. 2011 [15] | ≥1 positive BC for VRE that met the CDC definition, and received linezolid or daptomycin for ≥3 days | median 6.0, range 3.7–8.8 mg/kg | median 3, range 0–7 days | median 4, range 0.5–4 mg/L | CCI 4.6 ± 2.48 | APACHE II mean 15, range 6–33 |  | 600 mg q12h | median 2, range 0–11 days | median 2, range 0.5–2 mg/L | CCI 5.1 ± 2.6 | APACHE II mean 18, range 5–31 |
| McKinnell et al. 2011 [16] | CDC criteria for nosocomial BSI with VRE, and received linezolid or daptomycin for ≥3 days | NR | mean 2.25 ± 1.5 days | NR | CCI 4.6 ± 2.8 | NR |  | NR | mean 2.38 ± 1.7 days | NR | CCI 5.0 ± 2.8 | NR |
| Chou et al. 2012[17] | ≥2 separate positive BC for VRE or 1 positive BC with clinical features compatible with sepsis | NR | mean 3.5 ± 1.6 days | NR | CCI 4.6 ±3.2 | Pitts score 3.9 ± 2.5 |  | NR | mean 3.7 ± 1.3 days | NR | CCI 4.3 ± 2.3 | Pitts score 3.4 ± 2.9 |
| Lu et al. 2012 [24] | ≥1 positive BC for VRE with fever (body temperature ≥38°C) | NR | NR | All susceptible (≤4 mg/L) | NR | NR |  | NR | NR | All susceptible (≤2 mg/L) | NR | NR |
| Twilla et al. 2012 [18] | ≥1 positive BC for VRE, and received linezolid or daptomycin for ≥5 days | Mean 6.1, median6, range 3.4–10.4 mg/kg | mean 1.8 ± 1.5 days | All susceptible, range 0.25–4 mg/L | NR | NR |  | 600 mg q12h | mean 2.3 ± 1.8 days | All susceptible, but one >4 mg/L | NR | NR |
| Barbour et al. 2013 [19] | ≥1 positive BC for VRE with fever (body temperature ≥100.5 °F), and received linezolid or daptomycin for ≥2 days, neutropenia cancer patients | mean 6.4 mg/kg | Mean 52 hours | 3 isolates > 4mg/L | NR | NR |  | 600 mg q12h | Mean 43 hours | All susceptible | NR | NR |
| Furuya et al. 2005 [30] | VREB | NR | NR | NR | NR | NR |  | NR | NR | NR | NR | NR |
| El-Lababidi et al. 2007 [31] | VREB | NR | NR | NR | NR | NR |  | NR | NR | NR | NR | NR |
| Dubrovskaya et al. 2008 [32] | VREB, and received linezolid or daptomycin for ≥2 days | median 6, range 4-9 mg/kg | NR | NR | NR | APACHE II 19 ± 5 |  | NR | NR | NR | NR | APACHE II 21 ± 7 |
| Marion et al. 2008 [33] | VREB, febrile neutropenia | 6 mg/kg | NR | NR | NR | NR |  | 600 mg q12h | NR | NR | NR | NR |

*^a^* Time to initiation of antibiotics which was determined from collection of the first blood culture that subsequently grew VRE to the start date of antibiotics, measured in days

APACHE, Acute physiology and chronic health evaluation; BC, blood culture; BSI, bloodstream infection; CCI, Charlson comorbidity index; CDC, Centers for Disease Control and Prevention; HSCT, hematopoietic stem cell transplantation MIC, minimum inhibitory concentration; NR, not reported; VRE, vancomycin-resistant enterococci; VREB, vancomycin-resistant enterococcal bacteremia
