# Supplementary figures and images for: Daptomycin versus linezolid for treatment of vancomycin-resistant enterococcal bacteremia: systematic review and meta-analysis
Source: BMC Infect Dis. 2014 Dec 13;14:687. doi: 10.1186/s12879-014-0687-9 (PMC4269951; doi:10.1186/s12879-014-0687-9)

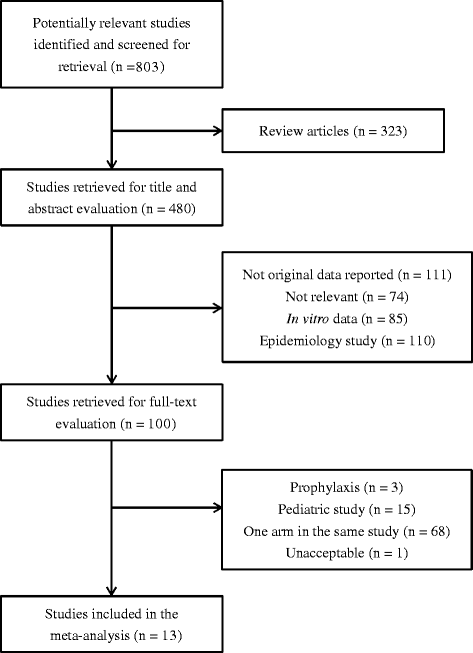

Supplement: Supplementary file 2 — Authors’ original file for figure 1 [file 12879_2014_687_MOESM2_ESM.gif]

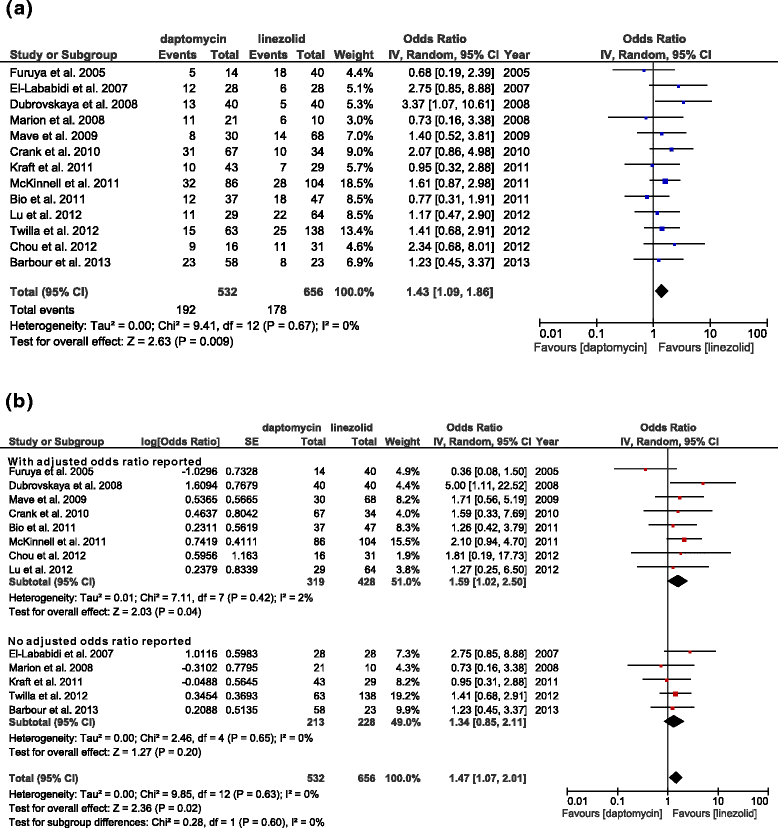

Supplement: Supplementary file 3 — Authors’ original file for figure 2 [file 12879_2014_687_MOESM3_ESM.gif]

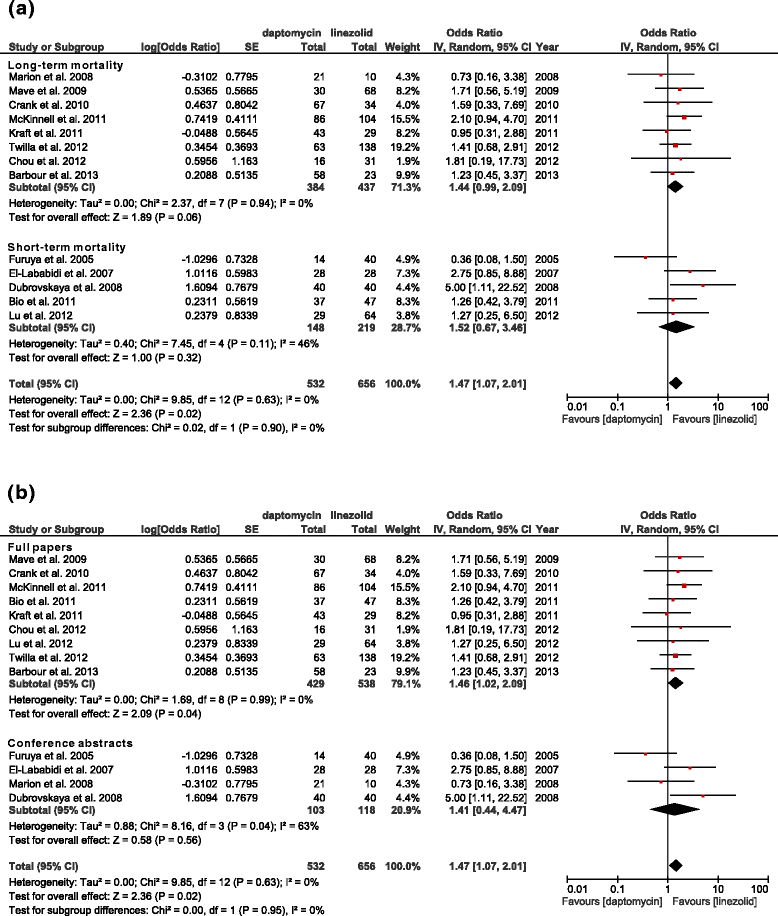

Supplement: Supplementary file 4 — Authors’ original file for figure 3 [file 12879_2014_687_MOESM4_ESM.gif]

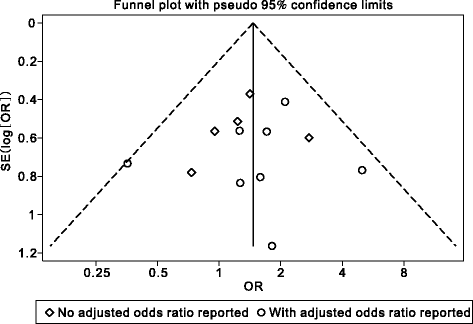

Supplement: Supplementary file 5 — Authors’ original file for figure 4 [file 12879_2014_687_MOESM5_ESM.gif]

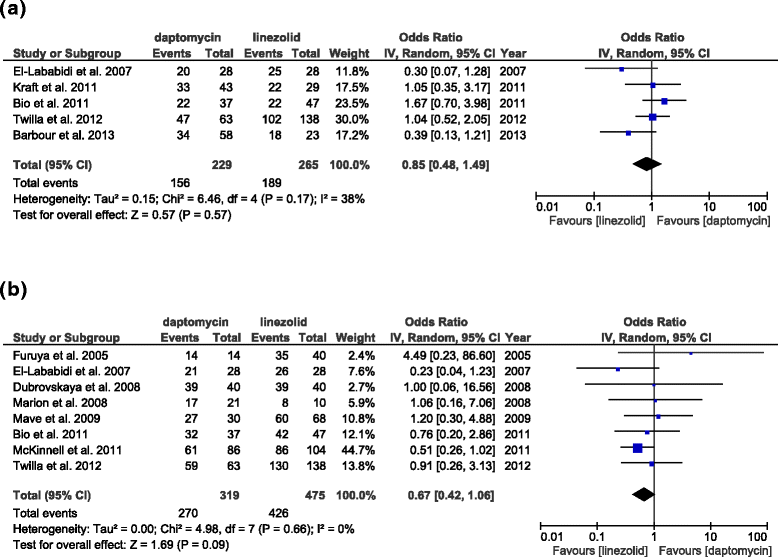

Supplement: Supplementary file 6 — Authors’ original file for figure 5 [file 12879_2014_687_MOESM6_ESM.gif]

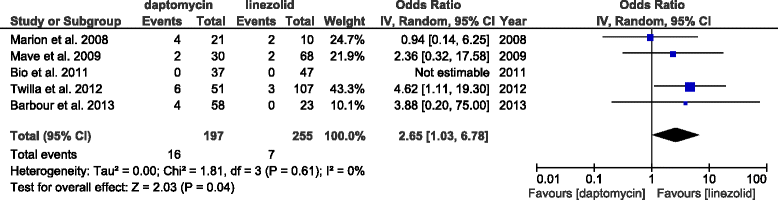

Supplement: Supplementary file 7 — Authors’ original file for figure 6 [file 12879_2014_687_MOESM7_ESM.gif]
